# Supplementary material for: Real-time label-free imaging of living crystallization-driven self-assembly
Source: Nat Commun. 2025 Mar 18;16:2672. doi: 10.1038/s41467-025-57776-9 (PMC11920093; doi:10.1038/s41467-025-57776-9)
Supplement: Supplementary file 1 — Supplementary Information [file 41467_2025_57776_MOESM1_ESM.pdf]

Supplementary Information

Real-time label-free imaging of living crystallization-driven self-assembly

Yujie Guo[†1], Tianlai Xia[†2], Vivien Walter[3], Yujie Xie[2], Julia Y. Rho[2], Laihui Xiao[2], Rachel K. O'Reilly[*2], and Mark I. Wallace[*1]

[1]Department of Chemistry, King's College London, London, SE1 1DB, UK
[2]School of Chemistry, University of Birmingham, Edgbaston, Birmingham, B15 2TT, UK
[3]Department of Engineering, King's College London, London, WC2R 2LS, United Kingdom

[†]These authors contributed equally to this work.
[*]Corresponding Author: r.oreilly@bham.ac.uk, mark.wallace@kcl.ac.uk.

Supplementary Methods

1.1 Kinetic model for 1D fiber growth

The model used for 1D fiber formation has been previously reported [1]. The length evolution of fiber in a CDSA process can be described as follow:

$$L(t) = \frac{1}{N_{\mathrm{agg}/L}} \frac{[U]_0}{[S]_0}(1 - e^{-2r[S]_0 t}), \tag{1}$$

where L_{t} is the average length of the fiber measured at time t, N_{agg} represents the linear aggregation number, $[U]_0$ is the initial unimer concentration, $[S]_0$ is the initial seed concentration and r is the rate constant. The length evolution as the function of all data was then fitted to Supplementary Eq. 1 using Scipy.optimize [2] and the rate constants can be extracted.

1.2 Kinetic model for 2D platelet growth

The model used for the 2D platelet growth study was established previously [3]. The overall rate equation is described by Supplementary Eq. 2:

$$\frac{dN_{\mathrm{t}}}{dt} = -k[4\pi B n_{\mathrm{a}}^2 (N_0 - N_{\mathrm{t}})]^{\frac{1}{2}} N_{\mathrm{t}}, \tag{2}$$

where N_{t} is the number of unimer molecules remaining in the reaction at time t, k is the overall rate constant, N_0 is the initial number of unimers, n_{a} is the number of unimers per unit platelet perimeter and B is the contribution of one unimer to the area of the platelet. The integration of Supplementary Eq. 2 gives the following result:

$$N_{\mathrm{t}} = \frac{2N_0}{\cosh\left(2n_a\sqrt{\pi B N_0}\,(C_1 - kt)\right) + 1}. \tag{3}$$

By setting t to 0 and N_{t} to N_0, the integration constant (C_1) can be removed. To further simplify the equation, an effective rate constant (k') was then introduced and can be expressed as:

$$k' = 2n_a\sqrt{\pi B N_0} \times (-k). \tag{4}$$

Supplementary Eq. 3 can be further expressed as:

$$N_{\mathrm{t}} = \frac{2N_0}{\cosh(k't) + 1}. \tag{5}$$

The contribution of one unimer to the area of the platelet (B) can be calculated from:

$$B = \frac{A_{\mathrm{final}} \times N_{\mathrm{seed}}}{N_0}, \tag{6}$$

where given A_{final} is averaged final platelet size, and N_{seed} is the number of seeds. Via this parameter, the number of consumed unimers (N_0 - N_{t}) is linked to platelet size (A_{t}):

$$A_{\text{t}} = \frac{B \cdot (N_0 - N_t)}{N_{\text{seed}}} = \frac{BN_0}{N_{\text{seed}}}\left(1 - \frac{2}{\cosh(k't) + 1}\right). \tag{7}$$

The size evolution as the function of all data was then fitted to Supplementary Eq. 7 using Scipy.optimize [2] and with rate constants can be extracted.

1.3 Kinetic data fitting for 1D fiber formation

After image segmentation, the length evolution of individual fibers is determined. To extract kinetic information, these data were fitted to Supplementary Eq. 1 using scipy.optimize [2], which produces a rate constant for each fitting. Initial unimer and seeds concentrations are known, and fixed during the fitting. The linear aggregation number (N_{agg}) was not fixed, the fitting was conducted based on the initial guess of N_{agg} equals to 3.5 chains nm^{-1}, which is reported linear aggregation number of poly(ferrocenyldimethylsilane)-*b*-(polydimethylsiloxane) [1].

1.4 Kinetic data fitting for 2D platelet formation

With the averaged area of platelets at each time point extracted, the evolution of platelet area as the function of reaction time can be plotted. To extract kinetic information, kinetic data fitting was conducted via an in-house Python script. The size evolution data was fitted to Supplementary Eq. 7 using scipy.optimize [2], which produces a rate constant for each fitting. To conduct the fitting, the initial number of unimer (N_0) and the number of seeds (N_{seed}) can be calculated from initial unimer and seed concentrations and fixed during the fitting. In addition, the contribution of one unimer molecule to the area of the platelets (B) is calculated from Supplementary Eq. 6 and fixed during the fitting.

1.5 Imaging Protocol

In this study, ‘real-time’ refers to the continuous observation of the formation process of CDSA assemblies, initiated by the self-assembly of block copolymers onto surface-immobilized seeds, which subsequently develop into 1D or 2D structures.

1. Sonication of polydisperse PCL_{45}-*b*-$PDMA_{348}$ fibers yielded short uniform crystalline seed solution.

2. To coat seeds onto the glass coverslip, 50 µL of 2.51 nM (0.1 µg mL^{-1}) seed solution was spin-coated onto the cleaned coverslip twice (3200 rpm for 50 s, followed by 4000 rpm for 30 s). A cleaned plastic spacer was then placed onto the seed-coated coverslip to form a reaction chamber.

3. The reaction chamber was then placed above the objective with the objective being adjusted to focus on the surface and ready for data collection.

4. Unimer stock solutions were diluted in methanol to achieve the targeted concentration and added into the reaction chamber (seed-coated surface). iSCAT imaging was initiated immediately upon application of the unimer mixtures.

5. iSCAT allows fast data acquisition with sub-millisecond temporal resolution, which allows the monitoring of the entire CDSA process.

1.6 In situ iSCAT monitoring of CDSA process

Image analysis for both 1D fibers and 2D platelets was conducted following the same protocol. Python scripts developed in-house were first used, with the following steps:

1. Image stacks were first cropped to remove the non-laser illumination area.

2. Dark counts subtraction was conducted by subtracting each frame by a frame recorded under the same conditions without laser illumination.

3. Laser spatial intensity fluctuations were suppressed by dividing each frame by its own modal pixel value.

4. Background correction is a common step in iSCAT data processing used to eliminate systematic artifacts. In dynamic detection experiments, the background can be obtained by calculating the temporal median of each pixel across a stack of images, captured before the appearance of the object of interest [4–6]. In our case, stacks (Supplementary Fig. 1A) were subtracted by a background corresponding to the median-average of around 10 frames corresponding to the image area prior to platelet growth (Supplementary Fig. 1B), which generates the background corrected result as shown in Supplementary Fig. 1C.

5. Frame averaging was conducted on some of the experiments (the details of averaging have been stated in each experiment).

6. A binary mask was created from a low-pass Gaussian filtered ($\sigma = 2$ px) replica of the image and used to isolate individual particles as shown in Supplementary Fig. 1D.

7. After setting the measurement scale, particle parameters, such as area, long/short axis length, and aspect ratio were then collected using the built-in 'Analyze Particle' function in Fiji [7].

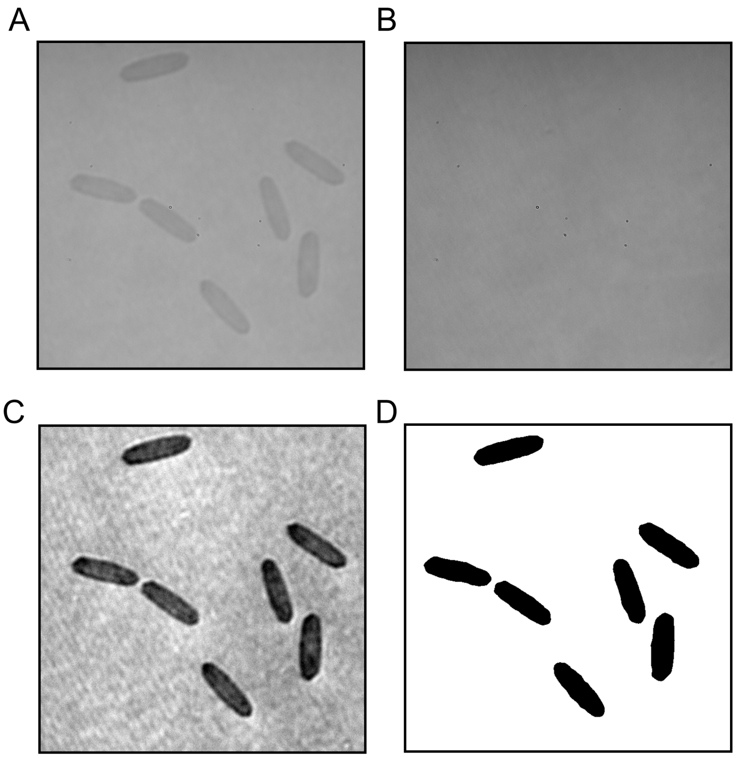

Supplementary Figure 1: **Schematic illustration of CDSA data process.** **(A)** Raw data. **(B)** Background generated from the median-average of 6 frames corresponding to the image area prior to platelet growth. **(C)** Data after background subtraction. **(D)** Data after applying a binary mask.

1.7 Assessment of noise reduction

To evaluate the effectiveness of our noise reduction method, we utilized the data presented in Figs. 2E and F as a demonstration. As shown in Supplementary Figs. 2A and B, the raw stack is characterized by high spatial and temporal variability, likely due to noise and artifacts. As displayed in Supplementary Figs 2C and D, following the protocol introduced above, the preprocessed data is significantly cleaner, with more uniform standard deviation and reduced fluctuations, improving its suitability for further analysis.

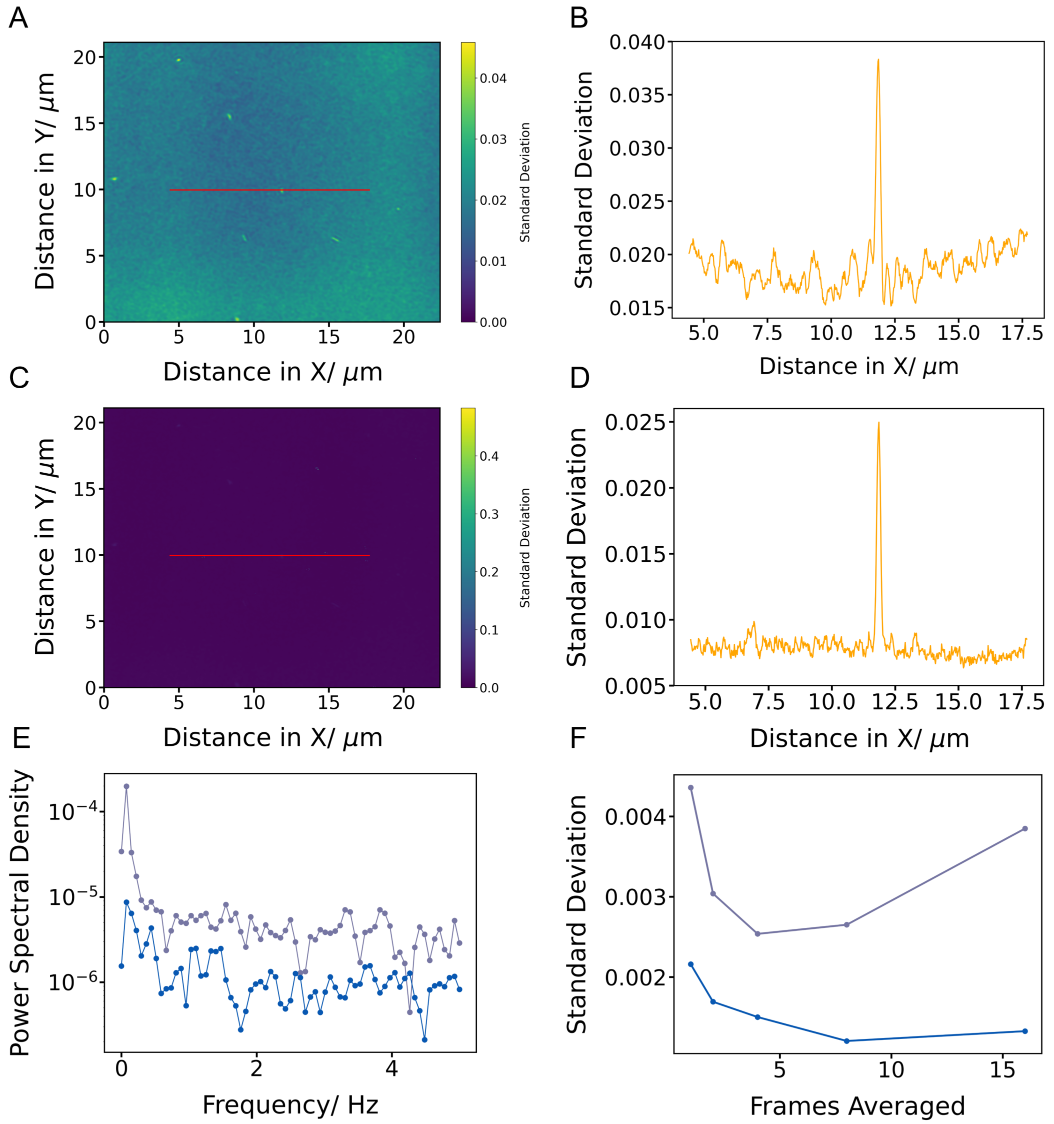

Supplementary Figure 2: **Assessment of noise reduction.**
(A) Temporal standard deviation map of the raw image stacks. **(B)** Cross-sectional profile of the temporal standard deviation drawn in **A**. **(C)** Temporal standard deviation map of the image stacks after preprocessing. **(D)** Cross-sectional profile of the temporal standard deviation drawn in **C**. **(E)** Comparison of power spectral density analysis for raw (purple) and preprocessed (blue) data. **(F)** Standard deviation of contrast as a function of the number of frames averaged together for raw (purple) and preprocessed (blue) data. This data corresponds to data shown in Figs. 2E and F.

1.8 Instrument calibration and contrast extraction

Gold nanoparticles with known diameters were chosen as standards for instrument calibration. A calibration curve was plotted based on the sizes of the gold nanoparticles

and their corresponding contrasts. Detailed results can be found in our recent publication [8].

To extract contrast information from sub-diffraction objects, the following protocol is followed:

1. The same procedures for image pre-processing, including cropping, dark counts subtraction, spatial intensity fluctuation removal, and background correction, were followed as described above.

2. To convert image intensities to contrast values, the background corrected stacks were then background-normalized by dividing these stacks by the background produced from median averaging. Particles were located using the Python module TrackPy [9]. Particles were selected based on the following filtering conditions: the diameter of the circle used to detect and analyze spots was set to 21 pixels; a minimum separation between particles of $2\times$ this diameter was specified; the minimum integrated brightness was set to 0.01 and a threshold with the value of 0.001 was applied. TrackPy returns information including the position and the size of tracked objects. A region of interest (ROI) was then defined by using the particle position as an origin and its size as the diameter. Then within the ROI, the pixel value with the maximum absolute value was defined as the contrast of the corresponding particle.

Supplementary Figures

2.1 Supplementary Figure 3

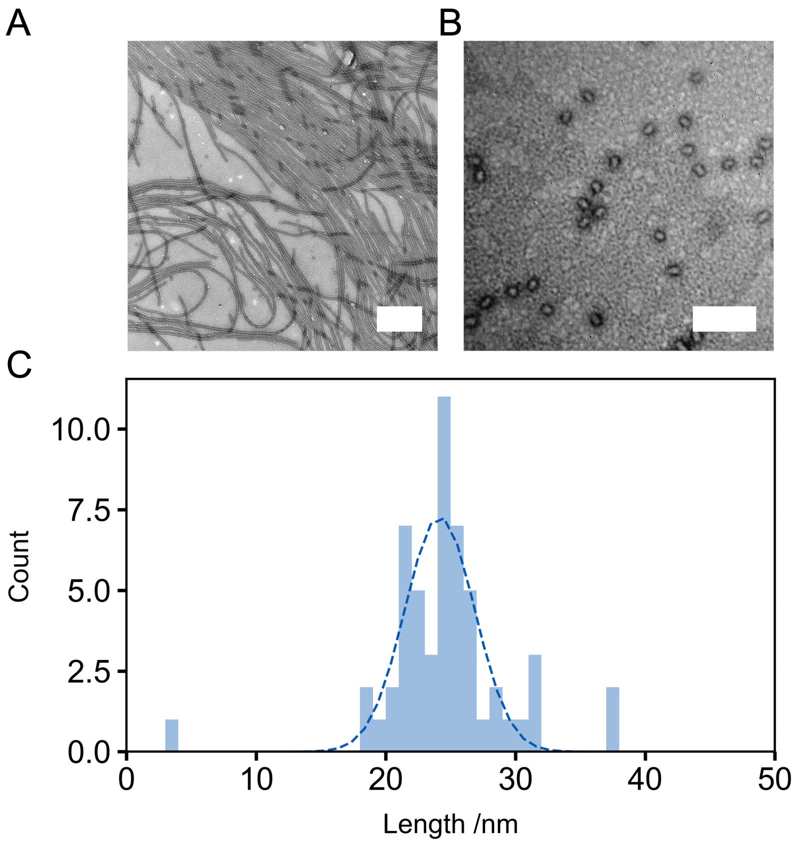

Supplementary Figure 3: **Seed characterization.** **(A)** TEM image of polydisperse fibers of PCL_{45}-*b*-$PDMA_{348}$ (scale bar: 1 μm). **(B)** TEM image of uniform PCL_{45}-*b*-$PDMA_{348}$ seeds (scale bar: 100 nm). **(C)** Size distribution of PCL_{45}-*b*-$PDMA_{348}$ seeds fitted with Gaussian distribution (mean: 24.6 nm, standard deviation: 4.9 nm).

2.2 Supplementary Figure 4

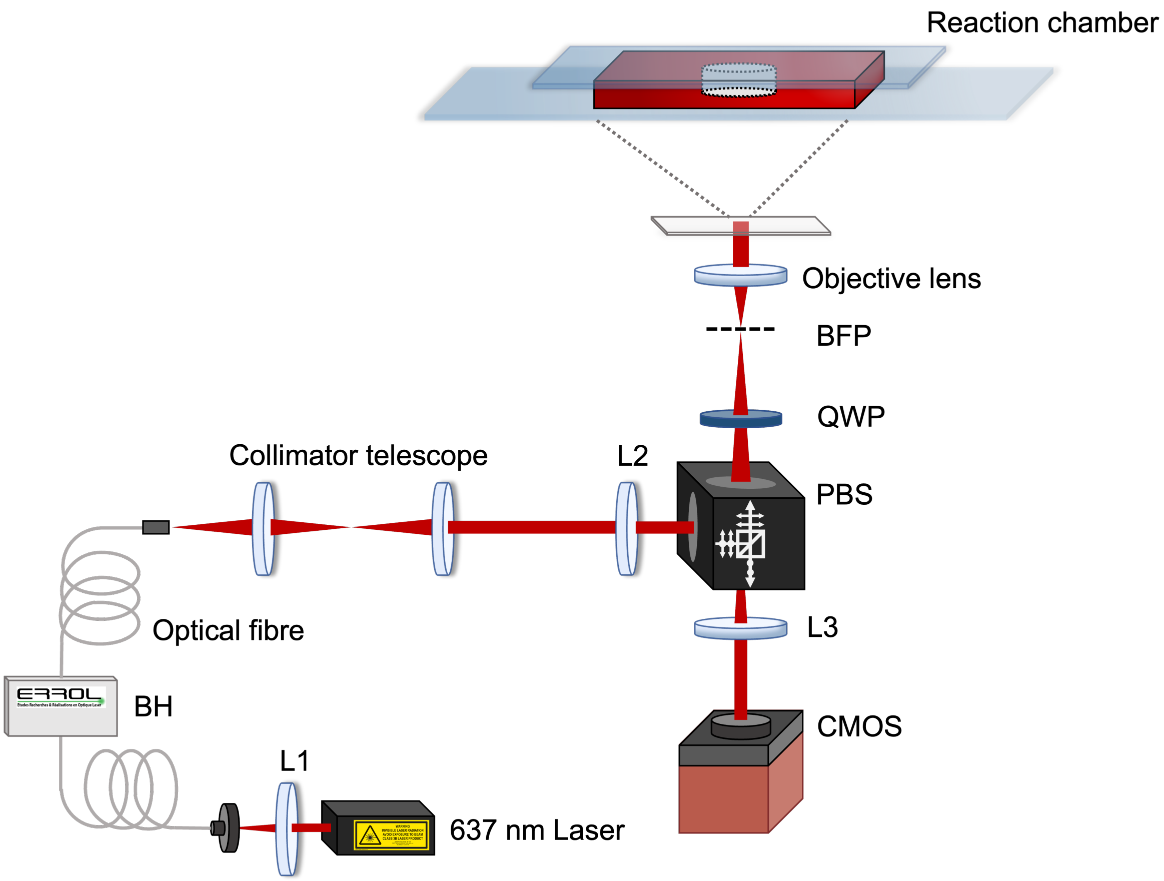

Supplementary Figure 4: **Details of apparatus.** iSCAT setup with a zoomed-in view of the reaction chamber. PBS, polarizing beam splitter; QWP, quarter-wave plate; BFP, back focal plane; CMOS, complementary metal oxide semiconductor sensor.

2.3 Supplementary Figure 5

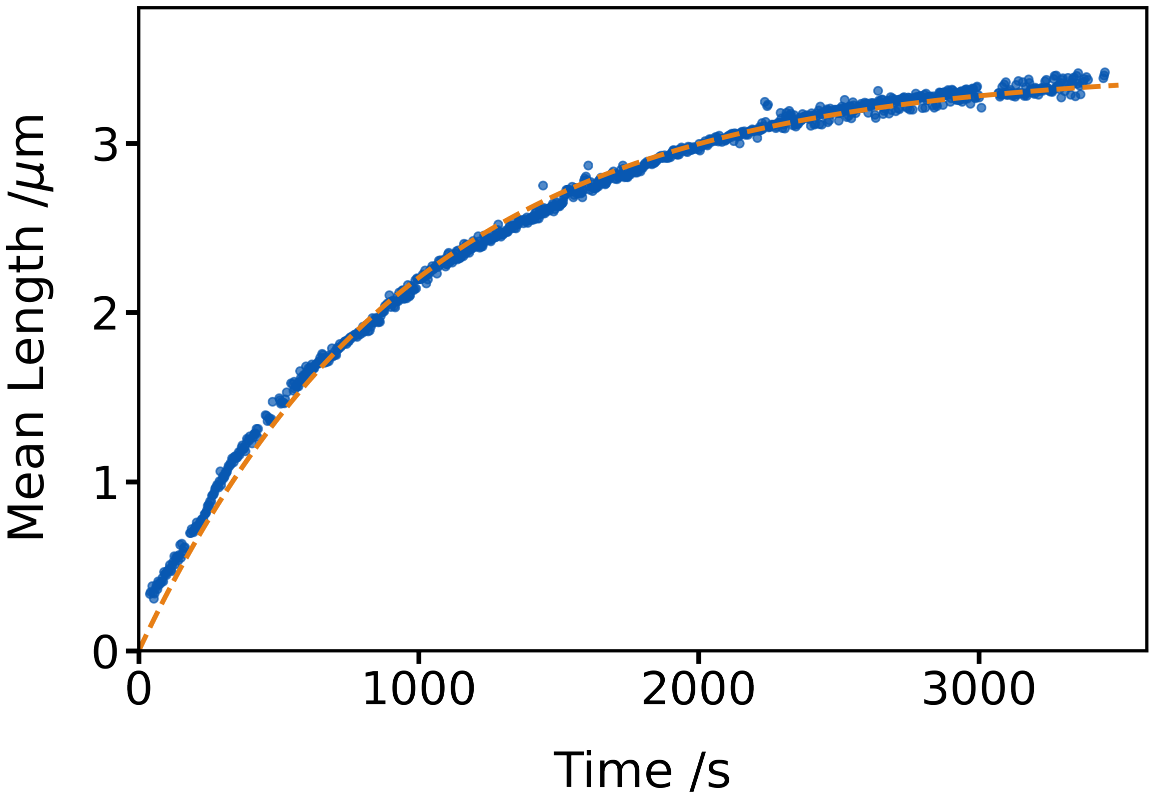

Supplementary Figure 5: **Lengths of PCL_{73}-*b*-$PDMA_{204}$ fibers as a function of time.** The mean length evolution was calculated from four fibers, as shown in Fig. 2B. The orange dashed line represents the fitted curve (rate constant: 0.0051 s^{-1}, N_{agg}: 4.86 chains nm^{-1}).

2.4 Supplementary Figure 6

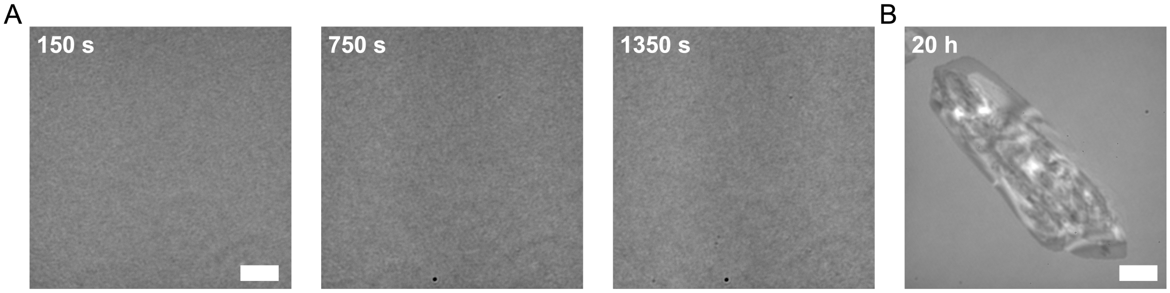

Supplementary Figure 6: **Control experiment: Reaction conducted in the absence of surface-coated seeds (A)** Montage of iSCAT images of CDSA conducted on blank coverslip (scale bar: 3 µm). 150 µL 0.35 µM (3.3 µg mL^{-1}) PCL_{45}:PCL_{45}-*b*-$PDMA_{348}$ unimer mixtures were added onto the blank surface followed by recording immediately. **(B)** Self-nucleation observed after prolonged reaction time to 20 h (scale bar: 3 µm).

2.5 Supplementary Figure 7

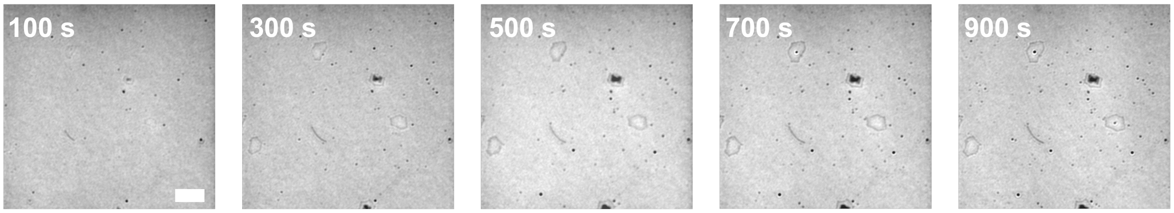

Supplementary Figure 7: **In situ iSCAT monitoring of poly(η-octalactone)-based CDSA platelets formation.** 50 µL of 2.51 nM (0.1 µg mL^{-1}) PCL_{45}-*b*-$PDMA_{348}$ seed solution was spin-coated onto the cleaned coverslip twice (3200 rpm for 50 s, followed by 4000 rpm for 30 s). 150 µL 1.67 µg mL^{-1} poly(η-octalactone):poly(η-octalactone)-*b*-poly(*N,N*-dimethylacrylamide) (POL_{55}:POL_{55}-*b*-$PDMA_{280}$) unimer mixtures (in ethanol) were added onto the seed-coated surface, followed by iSCAT imaging immediately. A laser power density of 4 µW µm^{-2} at 637 nm, an overall frame rate of 1 s^{-1}, and an exposure time of 520 µs were used (scale bar: 3 µm).

2.6 Supplementary Figure 8

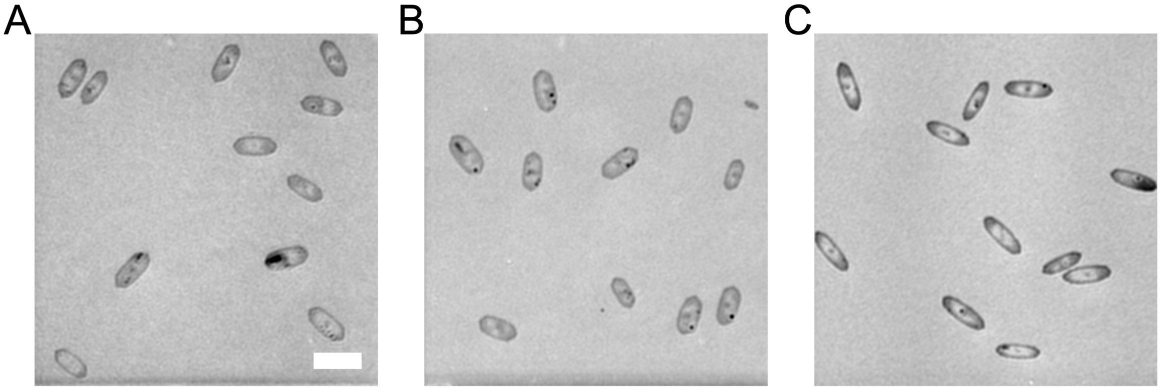

Supplementary Figure 8: **Impact of solvent on platelet formation.** **A** to **C**: Ethanol, isopropyl alcohol and methanol were used to prepare platelets, respectively. Methanol: water (30:70, v: v) solution was chosen as the imaging medium (scale bar: 3 µm). A laser power density of 4 µW μm^{-2} at 637 nm, an overall frame rate of 150 s^{-1}, and an exposure time of 400 µs were used, 100 frames were recorded and averaged.

2.7 Supplementary Figure 9

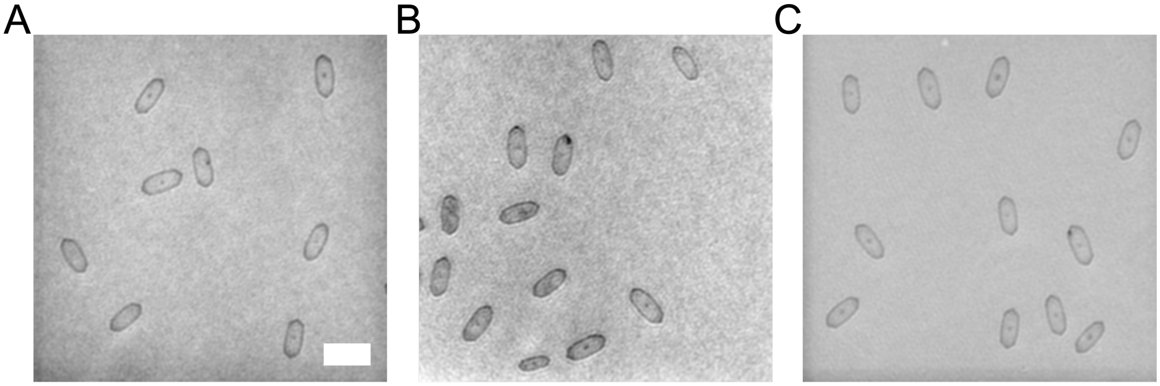

Supplementary Figure 9: **Impact of imaging medium on iSCAT imaging. A** to **C**: Platelets were prepared in ethanol and imaged in ethanol, isopropyl alcohol and methanol, respectively (scale bar: 3 µm). A laser power density of 4 µW μm^{-2} at 637 nm, an overall frame rate of 150 s^{-1}, and an exposure time of 400 µs were used. 100 frames were recorded and averaged.

2.8 Supplementary Figure 10

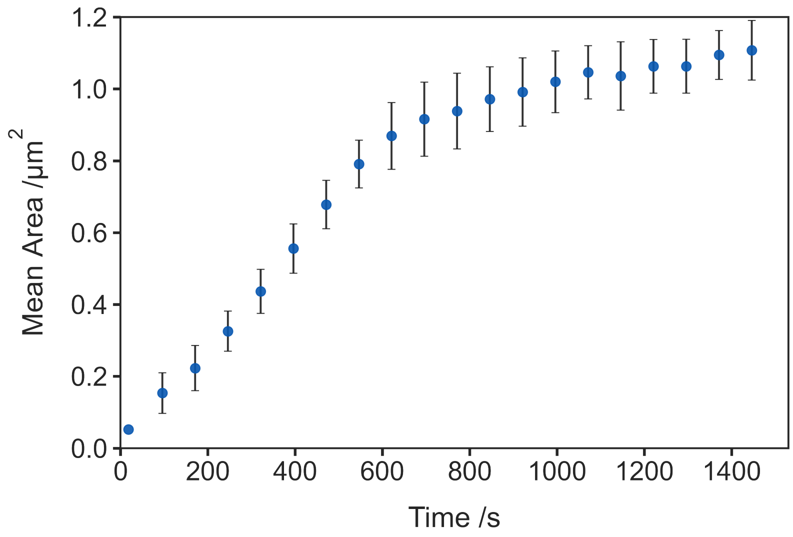

Supplementary Figure 10: **Mean area evolution of 40 platelets over time.** The mean area was calculated from 40 platelets (n=40) shown in Fig. 2D, with error bars representing the standard deviation.

2.9 Supplementary Figure 11

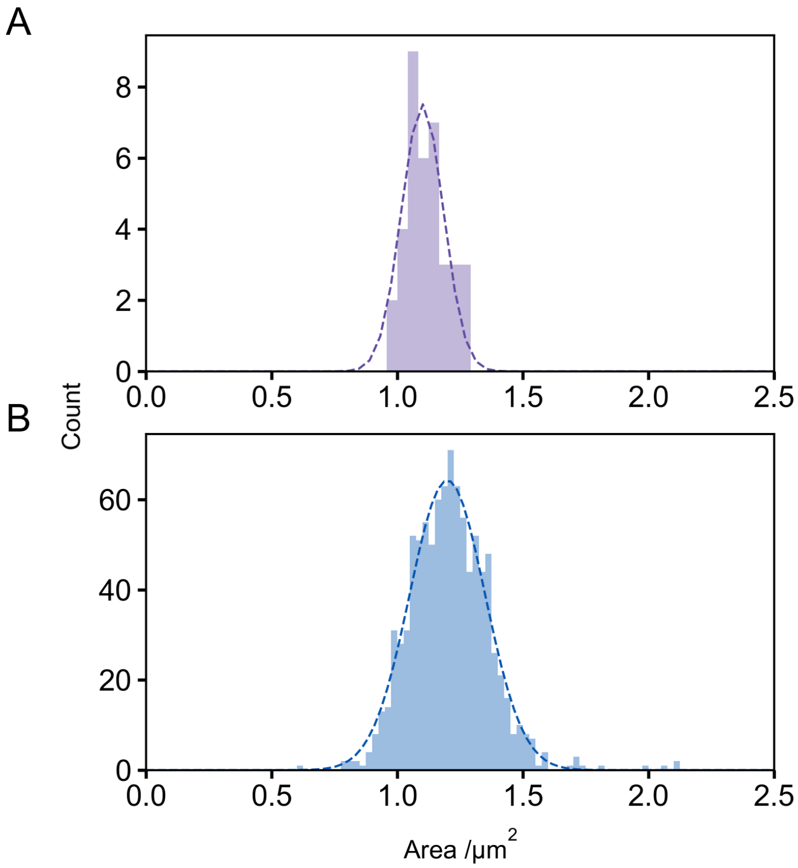

Supplementary Figure 11: **Comparison of platelet size distributions: End of in situ recording vs. subsequent sampling. (A)** Area distribution of platelets measured at the conclusion of each in situ recording session, corresponding to the area at time 1500s shown in Fig. 2D. **(B)** Area distribution of platelets sampled after the recording session at various sample positions. These distributions illustrate the consistency of platelet sizes recorded during the in situ sessions (area mean: 1.12 μm^2, standard deviation: 0.077 μm^2) with those obtained from subsequent sampling across different locations (area mean: 1.21 μm^2, standard deviation: 0.159 μm^2), dash lines represent Gaussian fittings.

2.10 Supplementary Figure 12

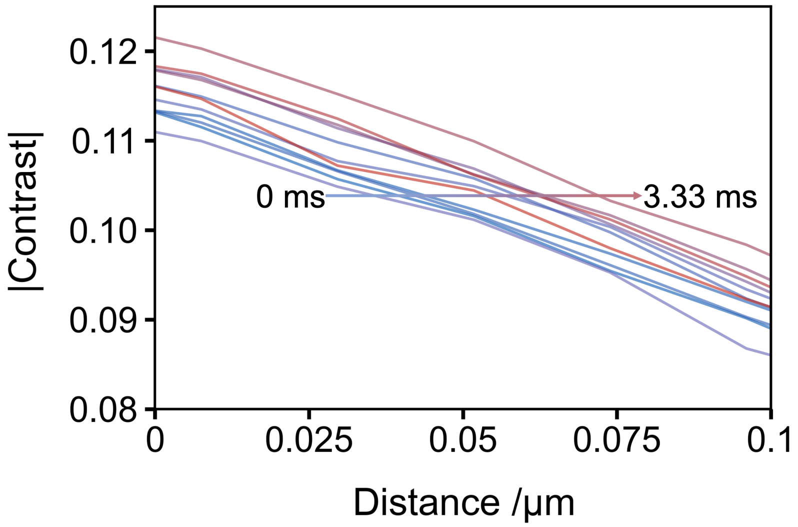

Supplementary Figure 12: **High temporal resolution imaging of individual platelet growth.** The contrast profile evolution of the cross-section (depicted in Fig. 2G) is reported with a temporal resolution of 0.33 ms over 3.33 ms.

2.11 Supplementary Figure 13

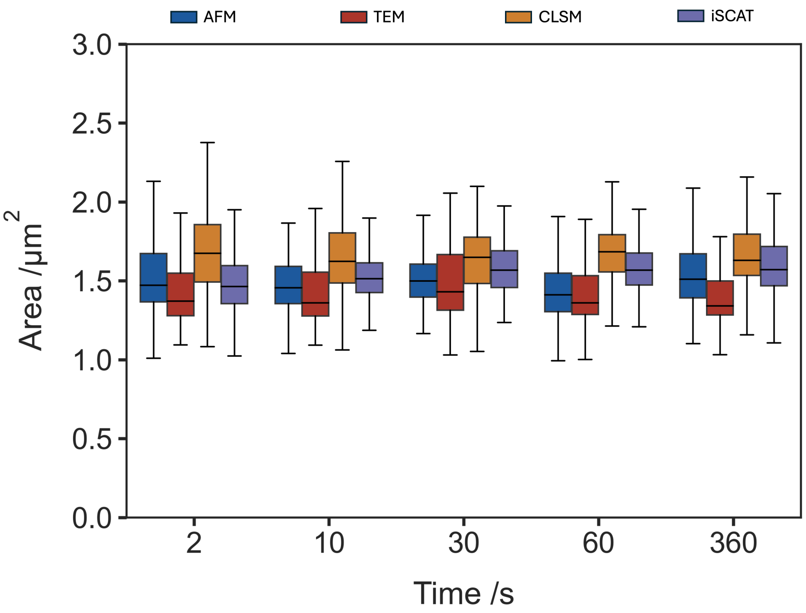

Supplementary Figure 13: **Comparison of 2D platelet characterization methods.** Same data from Fig. 3B with mean and standard deviation displayed (n=100).

2.12 Supplementary Figure 14

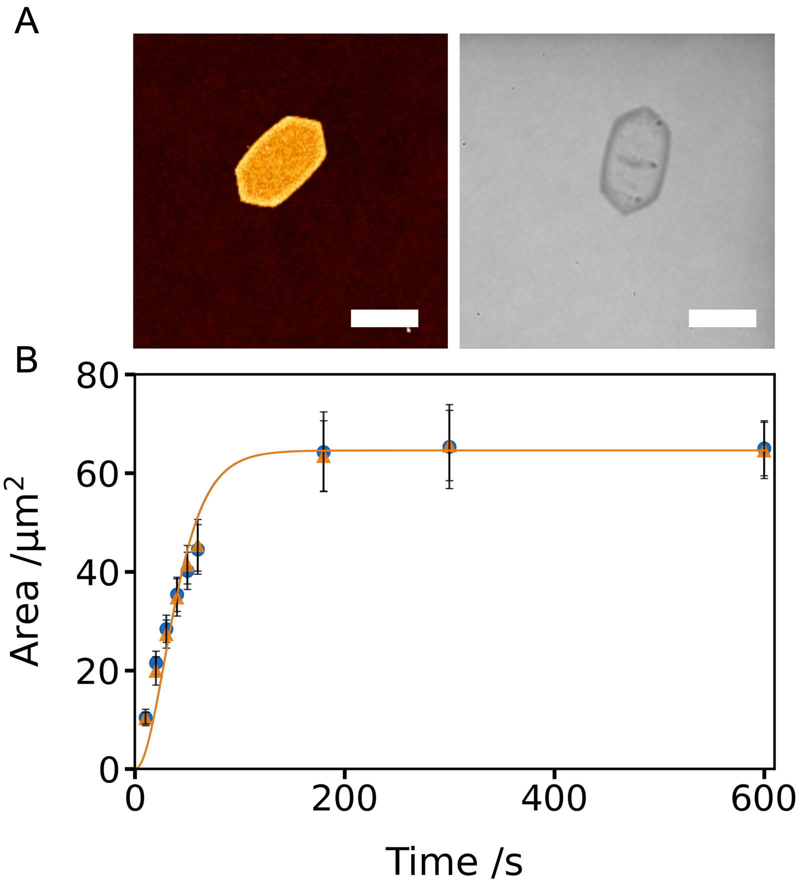

Supplementary Figure 14: **Comparative analysis of AFM and iSCAT in morphological and kinetic studies.** **(A)** AFM (top left, scale bar: 3 µm) and iSCAT (top right, scale bar: 3 µm) images of platelets collected at 10 seconds of reaction. The bulk reaction was conducted with final unimer and seed concentrations of 6.3 µM (60 µg mL^{-1}) and 0.87 nM (0.035 µg mL^{-1}), respectively. A 140 µL sample solution was withdrawn at predefined time intervals, quenched with deionized water, and spin-coated onto a glass coverslip for subsequent iSCAT and AFM analysis. **(B)** Kinetic study of platelet formation reported by AFM (blue circular markers) and iSCAT (orange triangular markers), with error bars representing the standard deviation. Around 100 platelets (n=100) were analyzed for each data point. The effective rate constants (k') extracted from the fitting for AFM and iSCAT are 0.0474 and 0.0473 s^{-1}, respectively.

2.13 Supplementary Figure 15

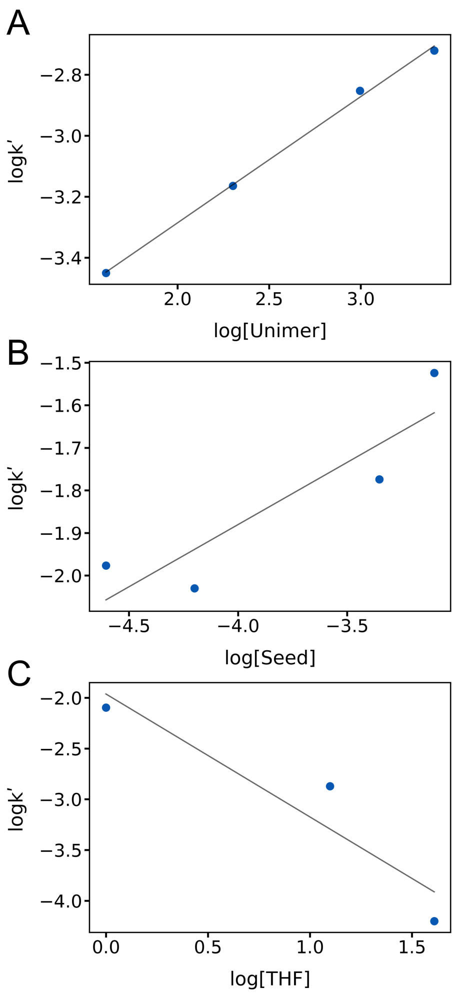

Supplementary Figure 15: **Reaction order analysis.** **(A)** Plot of rate constant versus unimer concentration, reaction order: 0.41. **(B)** Plot of rate constant versus seed concentration, reaction order: 0.29. **(C)** Plot of rate constant versus THF content, reaction order: -1.21.

2.14 Supplementary Figure 16

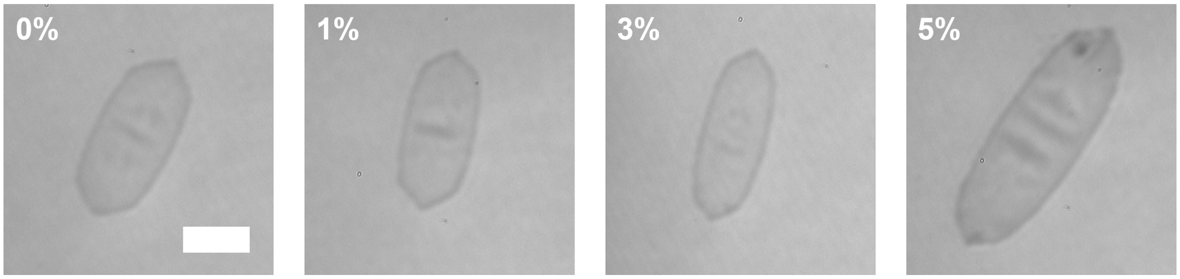

Supplementary Figure 16: **The effect of THF content on platelet morphology.** The volume fraction of THF in ethanol was varied from 0, 1, 3 to 5% (scale bar: 2 µm). Corresponds to the data in Figs. 4C, F and I (300 s).

2.15 Supplementary Figure 17

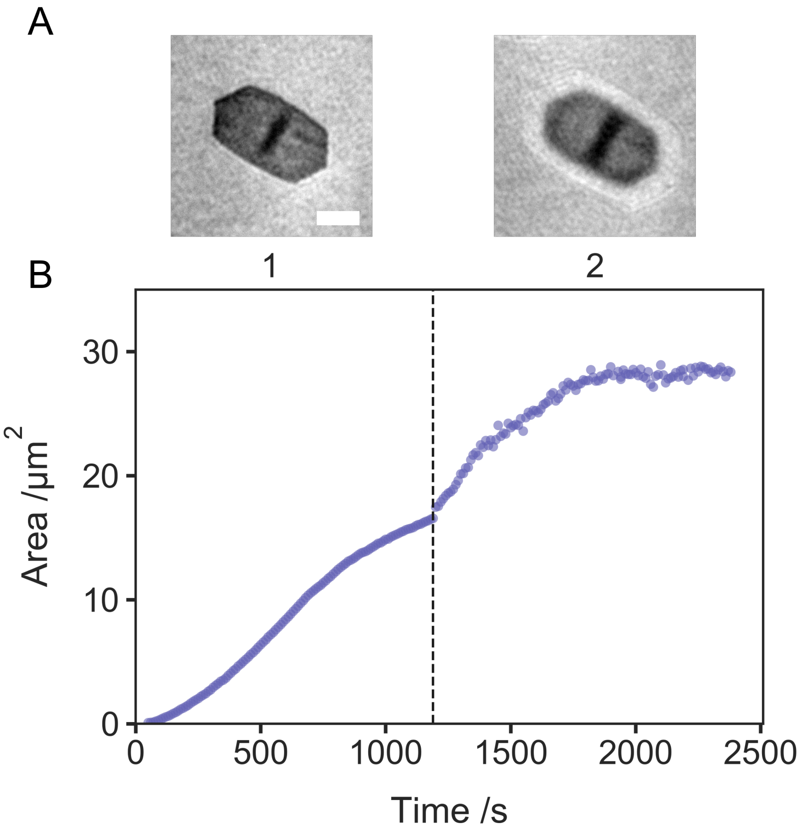

Supplementary Figure 17: **2-annulus platelet formation.** **(A)** Montage of iSCAT images of a 2-annulus platelet (scale bar: 2 µm). 50 µL of 2.51 nM (0.1 µg mL^{-1}) seed solution was spin-coated onto the cleaned coverslip twice (3200 rpm for 50 s, followed by 4000 rpm for 30 s). Annulus 1: 0.18 µM (1.67 µg mL^{-1}) PCL_{45}:PCL_{45}-*b*-$PDMA_{348}$ mixtures (PCL_{45} concentration: 0.15 µM, 0.83 µg mL^{-1}) in methanol. Annulus 2: 0.15 µM (0.83 µg mL^{-1}) PCL_{45}. A laser power density of 4 µW µm^{-2} at 637 nm, an overall frame rate of 1 s^{-1}, and an exposure time of 400 µs were used. **(B)** Size evolution of a 2-annulus platelet, with PCL_{45} concentration in each annulus kept the same. The data fluctuation in the area for the second annulus is because the contrast of the second annulus is relatively low, introducing errors during segmentation.

2.16 Supplementary Figure 18

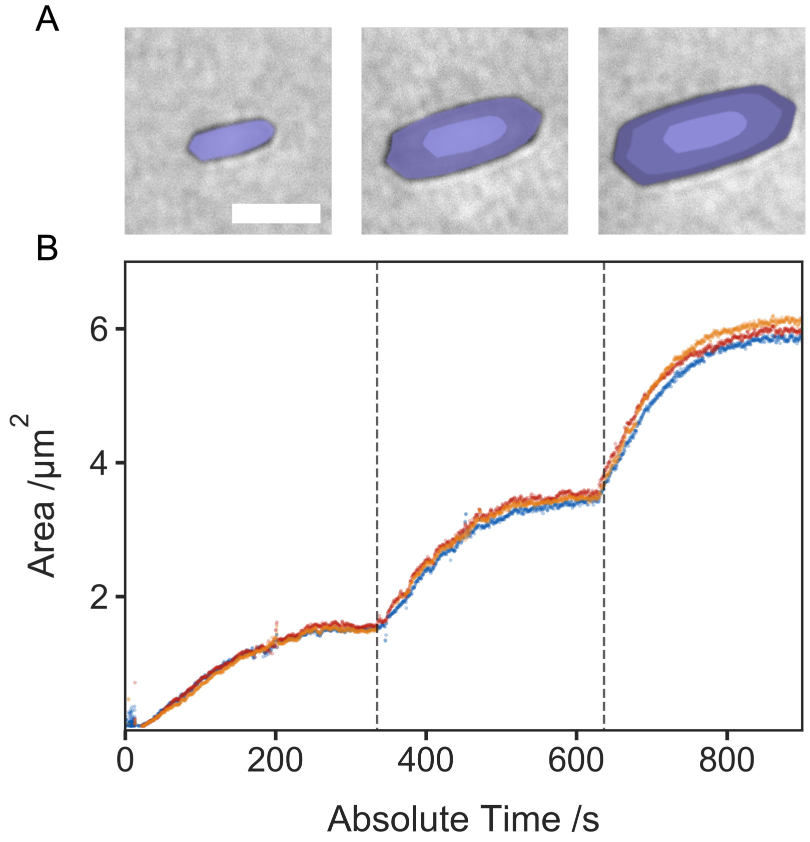

Supplementary Figure 18: **3-annulus platelets formation studied by iSCAT. (A)** Color-coded boundaries for three-annulus platelet shown in Fig. 5F. 50 µL of 2.51 nM (0.1 µg mL^{-1}) seed solution was spin-coated onto the cleaned coverslip twice (3200 rpm for 50 s, followed by 4000 rpm for 30 s), the PCL_{45}:PCL_{45}-*b*-$PDMA_{348}$ unimer mixtures were added sequentially with concentration of 0.44, 0.59 and 0.87 µM (4.2, 5.6 and 8.3 µg mL^{-1}) for each annulus. **(B)** Size evolution of three 3-annulus platelets. This data corresponds to the data shown in Fig. 5G.

2.17 Supplementary Figure 19

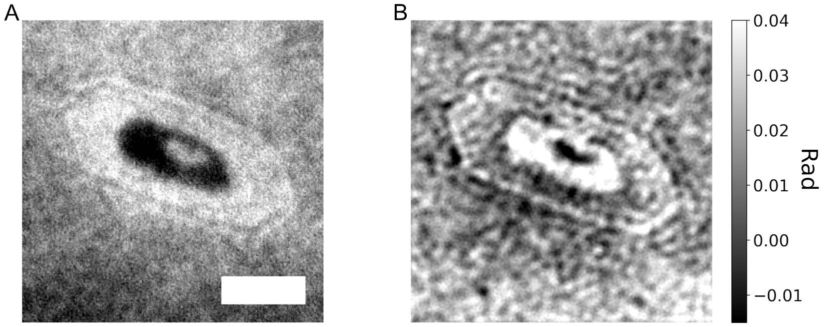

Supplementary Figure 19: **Phase imaging of two-annulus platelet. (A)** Raw iSCAT image of two-annulus platelet (scale bar: 2 µm). A laser power density of 4 µW µm^{-2} at 637 nm, an overall frame rate of 1 s^{-1}, and an exposure time of 400 µs were used. **(B)** Phase retrieved image of two-annulus platelets. An axial scan was performed with a step size of 0.1 µm.

Supplementary References

[1] C. E. Boott, E. M. Leitao, D. W. Hayward, R. F. Laine, P. Mahou, G. Guerin, M. A. Winnik, R. M. Richardson, C. F. Kaminski, G. R. Whittell, et al. Probing the growth kinetics for the formation of uniform 1d block copolymer nanoparticles by living crystallization-driven self-assembly. *ACS Nano*, 12(9):8920–8933, 2018.

[2] P. Virtanen, R. Gommers, T. E. Oliphant, M. Haberland, T. Reddy, D. Cournapeau, E. Burovski, P. Peterson, W. Weckesser, J. Bright, et al. SciPy 1.0: Fundamental algorithms for scientific computing in Python. *Nature Methods*, 17(3):261–272, 2020.

[3] C. Zhang, J. Lin, L. Wang, and L. Gao. 2D liquid-crystallization-driven self-assembly of rod–coil block copolymers: Living growth and self-similarity. *The Journal of Physical Chemistry Letters*, 13(26):6215–6222, 2022.

[4] K. Holanová, M. Vala, and M. Piliarik. Optical imaging and localization of prospective scattering labels smaller than a single protein. *Optics & Laser Technology*, 109:323–327, 2019.

[5] Y.-H. Lin, W.-L. Chang, and C.-L. Hsieh. Shot-noise limited localization of single 20 nm gold particles with nanometer spatial precision within microseconds. *Optics Express*, 22(8):9159–9170, 2014.

[6] M. Mazaheri *et al.* iSCAT microscopy and particle tracking with tailored spatial coherence. *Optica*, 11(7):1030–1038, 2024.

[7] J. Schindelin, I. Arganda-Carreras, E. Frise, V. Kaynig, M. Longair, T. Pietzsch, S. Preibisch, C. Rueden, S. Saalfeld, B. Schmid, *et al.* Fiji: An open-source platform for biological-image analysis. *Nature Methods*, 9(7):676–682, 2012.

[8] Y. Guo, V. Walter, S. Vanuytsel, C. Parperis, J. T. Sengel, E. E. Weatherill, M. I. Wallace, *et al.* Real-time monitoring and control of nanoparticle formation. *Journal of the American Chemical Society*, 145(29):15809–15815, 2023.

[9] D. B. Allan, T. Caswell, N. C. Keim, C. M. van der Wel, R. W. Verweij, *et al.* soft-matter/trackpy: Trackpy v0.5.0. *Zenodo*, 2021. `https://doi.org/10.5281/zenodo.4682814`.
