# Supplementary material for: Real-time label-free imaging of living crystallization-driven self-assembly
Source: Nat Commun. 2025 Mar 18;16:2672. doi: 10.1038/s41467-025-57776-9 (PMC11920093; doi:10.1038/s41467-025-57776-9)
Supplement: Supplementary file 2 — Description of Additional Supplementary Files [file 41467_2025_57776_MOESM2_ESM.pdf]

Description of Additional Supplementary Files

Supplementary Movie 1

Background-corrected movie of 1D fiber growth monitored by iSCAT microscopy (scale bar: 3 μm).

50 μL of 2.51 nM (0.1 μg/mL) seed solution was spin-coated onto a cleaned coverslip (3200 rpm for 50 s followed by 4000 rpm for 30 s). PCL_{73}-*b*-$PDMA_{204}$ unimer in chloroform were then diluted with methanol to achieve a final concentration of 0.06 μM (1.67 μg/mL). 150 μL unimer methanol solution was added onto the seed-coated surface and iSCAT observation started immediately. A laser power density of 4 μW μm^{-2} at 637 nm, a camera exposure time of 400 μs, and an overall time-lapsed frame rate of 3 s^{-1} were chosen. This movie corresponds to the data in Figs. 2A and B.

Supplementary Movie 2

Background-corrected movie of 2D platelet growth monitored by iSCAT microscopy (scale bar: 3 μm).

Plasma-cleaned coverslips were placed in 50 mL 0.002 μg/mL poly(ε-caprolactone)-*b*-poly(2-vinylpyridine) (PCL_{45}-*b*-$P2VP_{134}$) seed ethanol solution for 36 h. 150 μL of 0.35 μM (3.33 μg/mL) PCL_{45}:PCL_{45}-*b*-$PDMA_{348}$ unimer mixtures were then added onto the seed-coated surface followed by recording immediately. A laser power density of 4 μW μm^{-2} at 637 nm, a camera exposure time of 400 μs, and an overall time-lapsed frame rate of 1.5 s^{-1} were chosen. This movie corresponds to the data in Figs. 2C and D.

Supplementary Movie 3

Background-corrected movie of the early growth stage of platelets with sizes below the diffraction limit (scale bar: 3 μm).

50 μL of 2.51 nM (0.1 μg/mL) seed solution was spin-coated onto a cleaned coverslip twice (3200 rpm for 50 s followed by 4000 rpm for 30 s). PCL_{45}:PCL_{45}-*b*-$PDMA_{348}$ mixtures in THF were then diluted with methanol to achieve a final concentration of 0.35 μM (3.33 μg/mL). 150 μL of unimer methanol solution was added onto the seed-coated surface and iSCAT observation started immediately. A laser power density of 4 μW μm^{-2} at 637 nm, a camera exposure time of 400 μs, and an overall time-lapsed frame rate of 10 s^{-1} were chosen. This movie corresponds to the data in Figs. 2E and F.

Supplementary Movie 4

Background-corrected movie of platelet growth recorded with high speed (scale bar: 2 μm).

50 μL of 2.51 nM (0.1 μg/mL) seed solution was spin-coated onto the cleaned coverslip twice (3200 rpm for 50 s followed by 4000 rpm for 30 s). PCL_{45}:PCL_{45}-*b*-$PDMA_{348}$ mixtures in THF were then diluted with methanol to achieve final concentration of 0.29 μM (2.78 μg/mL). 150 μL unimer methanol solution was added onto the seed-coated surface and iSCAT observation started from the mid-stage of platelet growth. A laser power density of 24 μW μm^{-2} at 637 nm, a camera exposure time of 80 μs, and an overall time-lapsed frame rate of 3000 s^{-1} were chosen. This movie corresponds to the data in Figs. 2G and H.

Supplementary Movie 5
Background-corrected movie of a four-annulus platelet growth monitored by iSCAT microscopy (scale bar: 2 μm).
50 μL of 2.51 nM (0.1 μg/mL) seed solution was spin-coated onto the cleaned coverslip twice (3200 rpm for 50 s followed by 4000 rpm for 30 s). Annulus 1: 150 μL 0.18 μM (1.67 μg/mL) PCL_{45}:PCL_{45}-*b*-$PDMA_{348}$ methanol solution; Annulus 2: 150 μL 0.39 μM (2.08 μg/mL) PCL_{45} unimer methanol solution; Annulus 3: 150 μL 0.35 μM (3.33 μg/mL) PCL_{45}:PCL_{45}-*b*-$PDMA_{348}$ methanol solution; Annulus 4: 150 μL 0.77 μM (4.17 μg/mL) PCL_{45} unimer methanol solution. A laser power density of 4 μW μm^{-2} at 637 nm, a camera exposure time of 400 μs, and an overall time-lapsed frame rate of 1 s^{-1} were chosen. This movie corresponds to the data in Figs. 5B and C.

Supplementary Movie 6
Background-corrected movie of a three-annulus platelet growth monitored by iSCAT microscopy (scale bar: 2 μm).
50 μL of 2.51 nM (0.1 μg/mL) seed solution was spin-coated onto the cleaned coverslip twice (3200 rpm for 50 s followed by 4000 rpm for 30 s). PCL_{45}:PCL_{45}-*b*-$PDMA_{348}$ mixtures methanol solution were added sequentially with concentration of 0.44, 0.59 and 0.87 μM (4.2, 5.6 and 8.3 μg/mL) for each annulus. A laser power density of 4 μW μm^{-2} at 637 nm, a camera exposure time of 400 μs, and an overall time-lapsed frame rate of 0.5 s^{-1} were chosen. This movie corresponds to the data in Figs. 5F and G.
